# Gestational diabetes in Germany: Development of screening participation and prevalence

**DOI:** 10.25646/8325

**Published:** 2021-06-16

**Authors:** Lukas Reitzle, Christian Schmidt, Christin Heidemann, Andrea Icks, Matthias Kaltheuner, Thomas Ziese, Christa Scheidt-Nave

**Affiliations:** 1 Robert Koch Institute, Berlin Department of Epidemiology and Health Monitoring; 2 Institute for Health Services Research and Health Economics, German Diabetes Center, Leibniz Center for Diabetes Research at the Heinrich Heine University Düsseldorf, Düsseldorf; 3 Institute for Health Services Research and Health Economics, Center for Health and Society, Faculty of Medicine, Heinrich Heine University Düsseldorf, Düsseldorf; 4 German Center for Diabetes Research, Partner Düsseldorf, München-Neuherberg; 5 Scientific institute of specialized diabetologists, winDiab, Düsseldorf

**Keywords:** GESTATIONAL DIABETES, EPIDEMIOLOGY, SCREENING, PRENATAL CARE, DIABETES MELLITUS

## Abstract

Gestational diabetes mellitus (GDM) is an important risk factor for pregnancy complications. Since 2012, the Federal Joint Committee’s maternity directive recommends a two-step screening for GDM with a pre-test and subsequent diagnostic test if the pre-test is positive. This study analyses the implementation and development over time of GDM screening participation and prevalence in Germany. The data basis is the external inpatient obstetrics quality assurance documentation, which covers all births in hospital. Women with diabetes before pregnancy were excluded. The study defined women as GDM cases if the condition was documented in maternity records or if the ICD-10 diagnosis O24.4 was coded for inpatients at discharge and figures were determined for the years 2013 to 2018. As the documentation of screening tests has only been included in the data set since 2016, screening participation for the years 2016 to 2018 were estimated and evaluated based on the pre-test and/or diagnostic tests documented in maternity records. In 2018, the majority of all women who gave birth in hospitals had had a pre-test conducted (65.0%) or a pre-test and diagnostic test (18.2%) in line with the two-step procedure. A further 6.7% received a diagnostic test alone. GDM screening participation increased over time from 83.4% in 2016 to 89.9% in 2018. The prevalence of a documented GDM increased from 4.6% to 6.8% between 2013 and 2018. In 2018, this equates to 51,318 women with GDM. Reliably assessing the extent and causes of this development will require continuous analyses of screening implementation, documentation and changes in maternal risk factors.

## 1. Introduction

Gestational diabetes mellitus (GDM) is defined as a disorder of glucose tolerance occurring for the first time during pregnancy (Info box 1). GDM is one of the most common pregnancy complications and can have acute and long-term consequences for mother and child [1]. During pregnancy, GDM increases the risk of pre-eclampsia (a serious disease of the second half of pregnancy associated with high blood pressure, increased protein excretion in the urine and water retention), premature birth and caesarean section [2, 3]. Newborns of mothers with GDM are more likely to show malformations and high birth weight (macrosomia) [2, 3], which is associated with an increased incidence of birth injuries and shoulder dystocia [4]. In the long term, mothers with GDM have a significantly elevated risk of developing type 2 diabetes [5] and subsequently also increased mortality from cardiovascular disease [6].

**Info box 1**

**Gestational diabetes**

Gestational diabetes mellitus belongs to the group of diabetes mellitus related metabolic diseases. Gestational diabetes is defined as a glucose intolerance occurring for the first time during pregnancy and typically returning to normal after birth. Gestational must be distinguished from manifest type 1 or type 2 diabetes diagnosed for the first time during pregnancy. Hormonal changes during pregnancy lead to changes in insulin requirement, especially from the second trimester of pregnancy onwards. The decreased sensitivity of body cells to the hormone insulin (insulin resistance) can lead to an increase in blood sugar levels. Early diagnosis and treatment can reduce the pregnancy and birth related risks for mother and child resulting from gestational diabetes [3].

For this reason, Germany introduced general screening for gestational diabetes in 2012. According to the maternity directive of the Federal Joint Committee, screening is carried out in a two-step procedure and must be offered to pregnant woman between the 24th and 28th week of pregnancy [17]. First, a pre-test with 50g glucose (glucose challenge test, GCT) is carried out, which can be performed regardless of the time of day and recent food intake. If the blood glucose value in the preliminary test exceeds 135 mg/dL (7.5 mmol/L), a diagnostic test with 75g glucose (oral glucose tolerance test, oGTT) follows, for which the pregnant woman has to fast. If the pretest exceeds the value of 200 mg/dL (11.1 mmol/L) then the woman has a manifest diabetes mellitus. In line with international guidelines, German guidelines for gestational diabetes nonetheless recommend a diagnostic test directly, which is not covered by statutory health insurance [18].

According to estimates by the International Diabetes Federation, the prevalence of GDM ranges from 2% to over 30% worldwide [7]. Even within Germany, prevalence estimates of GDM vary considerably depending on the data source, study region and diagnostic criteria applied, and range between 5.1% and 13.2% [8–12]. International comparisons are complicated by diverging screening methods, diagnostic criteria and documentation systems [1, 13]. Consistently, an increase in the prevalence of GDM has been observed in most countries over the last decades [14]. Different factors have potentially contributed to this development, including changes to screening implementation and in the completeness of test result documentation [15] but also an increasing prevalence of important GDM risk factors such as obesity and advanced maternal age [1, 16].

Since 2012, pregnant women in Germany without pre-existing diabetes mellitus have been offered a two-step screening (Info box 2) for GDM based on the maternity directive of the Federal Joint Committee (G-BA) [17]. If gestational diabetes is diagnosed, the pregnant woman should make an appointment with a diabetologist, who will inform her about GDM and advise her on therapy options. The primary treatment consists of adjusting diet and exercise habits while regularly measuring blood glucose [18]. Insulin therapy is recommended for those women whose blood glucose levels do not return to normal with lifestyle changes.

In order to report the disease dynamics and determinants of diabetes mellitus in Germany on a recurring basis, a set of 40 indicators was defined within the framework of the diabetes surveillance at the Robert Koch Institute, which include risk factors, the disease frequency, care and the social impact of diabetes [19]. Within this framework GDM prevalence and screening participation are two core indicators, as GDM represents an important risk factor for developing type 2 diabetes later in life [20]. Since 2015, the Institute for Quality Assurance and Transparency in Health Care (Institut für Qualitätssicherung und Transparenz im Gesundheitswesen, IQTIG) administrates the inpatient quality assurance data for obstetrics, which are made available on request for research purposes within the framework of secondary data use since 2019. These contain the details from the maternity records of all women who gave birth in hospital and information on inpatient stay, including the diagnosis at discharge. Based on this data source, the present study estimates the development of screening participation and the prevalence of GDM in Germany over time. In addition, the implementation and results of two-step testing are evaluated in detail.

**Info box 2**

**Screening**

Screening is defined as the routine examination of persons without symptoms of disease for the presence of disease. The aim is to identify people at high risk of a particular disease and to diagnose the disease as early as possible [21]. The basic idea behind this is that starting treatment at an earlier stage of the disease improves the results of treatment. A well-known example is mammography screening, which aims to detect breast cancer as early as possible [22]. The disadvantages of screening that have been discussed are false positive findings and disease stages that do not necessarily require treatment, which in turn can lead to health burdens, unnecessary therapies and an unfavourable cost/benefit ratio [23].

## 2. Methodology

### 2.1 Obstetrics data

Data from quality assurance procedures pursuant to Section 136 of the German Social Code (Sozialgesetzbuch, SGB) V of the Federal Joint Committee (Gemeinsamer Bundesausschuss, G-BA) were used for this study. According to the G-BA’s directive on data-supported cross-facility quality assurance, all hospitals licensed under Section 108 of SGB V regularly submit measurement data on the quality of care with the aim of ensuring and promoting the quality of medical care [24]. In 2001, a corresponding quality assurance procedure was established for the field of obstetrics (becoming part of the quality assurance procedure perinatal medicine in 2021) covering all hospital births [11]. IQTIG currently compiles the data and ensures the quality assurance procedures. Secondary data use has been possible upon request and after approval by the G-BA for external applicants since 2019. Applicants receive the requested results in the form of aggregated data. The obstetrics dataset consists of two sub-datasets with information on mothers (16/1:M) and on newborn children (16/1:K). In addition to demographic information on pregnant women, the dataset includes information on the course of pregnancy, birth and the newborn child. Information on the course of pregnancy is mainly based on maternity record logs. Hospitals transmit the data to the IQTIG using a standardised documentation form with the data collected during birth at the hospital [25].

**Annex Figure 1:**
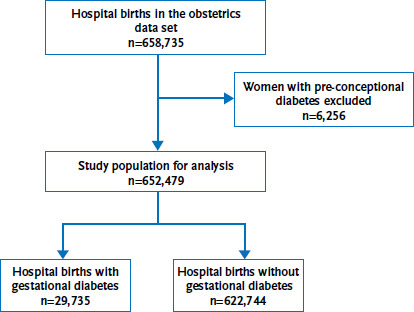
Flowchart for the analysis of gestational diabetes and the screening participation as an example for 2013 Source: Own figure

The present study relies on data collected during the reporting years 2013 to 2018. Women diagnosed with diabetes before becoming pregnant (pre-conceptional diabetes) and where this was documented in the maternity record at the first screening in catalogue A were excluded (Annex Figure 1). To assess completeness, the obstetric data were compared with the number of births published by the Federal Statistical Office [26]. Since the Federal Statistical Office only publishes the number of newborns, the total number of births was estimated using the number of live and stillborn children and the number of multiples for each year.

### 2.2 Definition of screening participation

The evaluation of screening tests was limited to the reporting years 2016 to 2018, as tests have only been included in the data set from 2016 onwards following a decision by the G-BA to document them in maternity records in April 2014 [27]. The information on pre-tests and the diagnostic tests is contained in catalogue B of the maternity record and registered under ‘Special findings during pregnancy’. The physician attending the pregnant woman enters in the maternity record whether a preliminary test and a diagnostic test were carried out (yes/no) and whether the test was abnormal (yes/no).

### 2.3 Definition of gestational diabetes

Cases of GDM were defined as GDM documented in the maternity record or the coding of GDM in the discharge diagnoses of the hospital stay at birth. In the maternity record, GDM is documented in catalogue B under ‘Special findings during pregnancy’ by the physician who made the diagnosis of GDM. Discharge diagnoses are coded according to the International Statistical Classification of Dis eases and Related Health Problems, 10th Revision, German Modification (ICD-10-GM [28]). GDM was assumed for the ICD-10 diagnosis O24.4.

### 2.4 Statistical analyses

All data used to calculate GDM screening participation and prevalence were provided in aggregated format by the IQTIG stratified by reporting year and maternal age using the following age groups: <20 years, 20 to 24 years, 25 to 29 years, 30 to 34 years, 35 to 39 years, 40 to 44 years and 45 years and older. To calculate GDM prevalence, a quotient was formed per age group and reporting year with the number of hospital births presenting maternal GDM according to the definition as numerator and all hospital births after excluding women with pre-conceptional diabetes as denominator. The screening participation was calculated analogously, excluding women with missing information on screening tests. For the screening participation, the results were analysed differentiated according to the test procedures performed (‘pre-test only’, ‘diagnostic test only’, ‘pre- and diagnostic test’ and ‘no test’). In addition, age-standardised values of GDM prevalence were calculated based on the mentioned age groups. The age distribution of the study population from the reporting year 2018 was used as the reference population (Table 1).

## 3. Results

### 3.1 Description of the study population

A comparison of the number of hospital births with the birth figures of the Federal Statistical Office shows a high degree of completeness. Depending on the reporting year, figures deviated between 2.5% and 3.6% (Annex Table 1). Deviations are owed to births outside the hospital as well as to women with pre-conceptional diabetes, a condition found in around 1% of the women who gave birth in hospital each year (Annex Figure 1). Birth numbers have increased since 2013 to more than 750,000 births in 2018 (Table 1). Over a third of mothers gave birth between the ages of 30 and 34. While the proportion of births in the 20 to 24 age group has decreased, the proportions in the 30 to 34 and 35 to 39 age groups have increased over time.

### 3.2 Gestational diabetes screening participation

**Figure 1:**
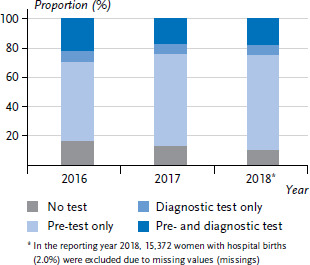
Development of the proportion of women with hospital births according to the test procedure used during pregnancy (n=2,243,518) Source: External inpatient quality assurance for obstetrics at IQTIG, own calculations

Figure 1 shows the proportion of women with a hospital birth for the years 2016 to 2018 who received a pre-test and diagnostic test, only a diagnostic test, only a pre-test or no test. For the reporting year 2018, no information was available for 2.0% of women (missings), with no differences in the age distribution of women with documented screenings. Over time, there was a decrease in the proportion of women who were not screened and a corresponding increase in the proportion with screening results to 89.9% in 2018. While the proportion of women who gave birth in hospital and had only a diagnostic test performed remained relatively constant, figures for women with only a pre-test increased over time.

**Figure 2:**
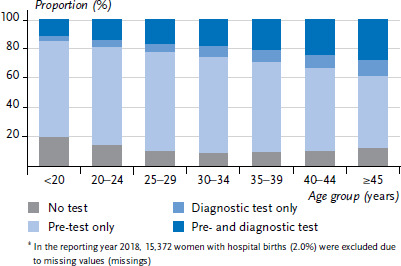
Proportions of women with hospital births in 2018^*^ by test procedure used in pregnancy and age at birth of child (n=737,401) Source: External inpatient quality assurance for obstetrics at IQTIG, own calculations

By age group, the highest figure for women who were not tested (19.2%) is found in the age group under 25 (Figure 2). Between the ages of 25 and 44, the proportion is relatively constant at around 10% and then increases slightly for women aged 45 and older. In the age groups under 35, two thirds of women with a hospital birth receive only a pretest, while only half of women aged 45 and older receive only a pre-test. The proportion of women who have both tests or only a diagnostic test increases significantly with age. Age-specific distribution patterns are constant over the reporting years (Annex Table 2), meaning that the decline in the proportion of women without a test cannot be attributed to a specific age group.

Maternity records document test procedures as well as test results. For the ‘pre-test only’ group, the pre-test result, for the other two tested groups the result of the diagnostic test was considered (Table 2). In the ‘pre-test only’ group, between 2016 and 2018, over 97% of the tested pregnant women consistently tested negative and thus were not affected by GDM. About 3% of the ‘pretest only’ group were positive and just under a quarter of them were also diagnosed as GDM. For the groups of pregnant women who received pre-test plus diagnostic test or only a diagnostic test, the proportion of positive tests increased from 25.7% to 37.6% and from 13.9% to 17.6% respectively between 2016 and 2018. For all three tested groups, the proportion of positive tests increases significantly with age, and is highest (56.6%) for women in the ‘pre-test plus diagnostic test’ group aged 45 and older (Annex Table 3).

### 3.3 Prevalence of gestational diabetes

Documented GDM prevalence figures show a continuous increase from 4.6% in 2013 to 6.8% in 2018 (Table 3). Combined with the simultaneous increase in the total number of births, this translates to an increase from 29,735 to 51,318 women with GDM in the observation period. The increase in GDM prevalence affects all age groups meaning that the age-standardised prevalences are only slightly higher.

Most women diagnosed with GDM have had both a pre-test and a diagnostic test performed. Thus, in the reporting year 2018, 75.4% of the women with GDM were in the group ‘pre-test and diagnostic test’ and 12.3% in the ‘diagnostic test only’ group. The remaining women received either only a pre-test (10.3%) or no test (2.0%). Between 2016 and 2018, there was a slight decrease in the group without documented testing from 3.5% to 2.0%, which was accompanied by an increase in the group ‘pre-test and diagnostic test’.

Comparing the proportion of women with a positive diagnostic test and with documented GDM shows that the proportion of women with a positive diagnostic test is higher. While 6.8% had a positive diagnostic test in 2016, this proportion increased to 7.9% in 2018, 1.5 and 1.1 percentage points higher than the proportion with documented GDM.

## 4. Discussion

This study is the first to estimate the development of the documented screening participation and prevalence of gestational diabetes in Germany using data from inpatient obstetric quality assurance. Most recently, 89.9% of pregnant women took part in screening and the proportion without testing has decreased significantly since 2016. Since the introduction of screening in 2012, there has been a steady increase in GDM prevalence and in 2018, 6.8% of women who gave birth in hospital had GDM documented in their maternity records.

Analyses of outpatient claims data from 2014/2015 already show that 80.8% of women were screened for GDM during pregnancy [12]. The present study suggests that this proportion has further increased over time and that screening also reaches women covered by private health insurance. In both studies, more than three quarters of pregnant women participated in the two-step screening. With age, the proportion of women who take both the pre-test and the diagnostic test increases significantly, which is because the age of the mother at birth is an important gestational diabetes risk factor. Only a small proportion of women receive a diagnostic test alone, which is somewhat higher in the present analysis (6.7% against 4.8%).

Estimates on the prevalence of gestational diabetes in Germany vary considerably depending on the data source, observation period and diagnostic criteria applied (Table 4). Population-based studies or cohort studies report a GDM prevalence of five to eight percent [8, 9, 29, 30] and are clearly above the estimates based on inpatient quality assurance data collected at the same time [11, 31]. However, the latter have increased significantly over the last few years. Analyses of statutory health insurance claims data provide higher GDM prevalence estimates [12, 32–34]. In line with this study, the available time series analyses show an increase in GDM prevalence over time. Reliable estimates of GDM prevalence are necessary to assess the extent and causes of this development and thus the potential for prevention.

For this reason, it is important to consider the influence of different data sources and diagnostic criteria with regard to an under- or overestimation of GDM prevalence. Except for the analysis of data from the Association of Statutory Health Insurance Physicians of Nordrhein (KV Nordrhein) [10], the study population of all estimates refers to women who have given birth and thus excludes pregnant women who have suffered miscarriages (Table 4). The study based on 2017 outpatient claims data examined the largest study population to date with 75% of all births, but this only included women covered by statutory health insurance that received continuous outpatient care [12]. Furthermore, case definitions in the studies also differed significantly. In claims data and inpatient quality assurance data, the prevalence of GDM is estimated on the basis of documented diagnoses [10, 12, 32, 33] or corresponding logs in maternity records [11, 31, 35] whereas survey and examination studies used measurement results to determine GDM prevalence [8, 9, 29, 30]. In claims data analyses, it is difficult to distinguish gestational diabetes from newly diagnosed manifest diabetes. For example, in approximately 1% of cases, in addition to gestational diabetes (ICD-10 diagnosis O24.4), manifest diabetes (ICD-10 diagnosis: O24.0–O24.3 or E10–E14) was also newly documented during pregnancy [10, 12]. These cases were only excluded in GDM prevalence estimate calculations in the analyses based on KV Nordrhein data [10]. Furthermore, in two studies that were based on claims data, a high proportion (44% and 33%, respectively) of women diagnosed with GDM received only a pre-test [12, 33], which only indicates GDM or diabetes if the result is highly abnormal [18, 36]. In the present study, this proportion is much lower at 10% to 11% (data not shown). This may have contributed to an overestimation of prevalence in claims data, leaving the magnitude of the discrepancy with the current analysis unexplained. An underestimation of prevalence by the present study cannot be ruled out either, as despite a documented positive diagnostic test result some women still did not receive a GDM diagnosis. The proportion of women with a positive diagnostic test result is 1 to 1.5 percentage points higher than GDM prevalence. More in-depth analyses should determine the extent to which this is due to incomplete documentation or also actually a diagnosis of new type 1 or type 2 diabetes.

Furthermore, subgroups of pregnant women with a particularly high or low GDM risk may not be being tested. An Austrian study concluded that women with a migration background took part in screening less frequently, but showed more frequent abnormal findings than women without a migration background [37]. The latter was also reported by a regional analysis of data from the AOK Berlin [32]. In addition, an analysis of hospital births in Bavaria showed that GDM prevalence is increased in socioeconomically deprived regions, characterised, for example, by higher unemployment and lower income [35]. The correlation was not evident before the introduction of general screening, so it can be assumed that especially women in regions with high social deprivation are reached by general screening. The inpatient quality assurance of obstetrics data set offers opportunities for further analyses regarding women who have not yet been reached by screening. In this case, regional differences and maternal risk factors for GDM can be specifically examined. A comparison between European countries is also difficult due to different diagnostic criteria [15, 38]. Countries with two-step GDM screening show lower prevalences than single test countries [39, 40]. Similar to the development in Germany, GDM prevalence over time is rising in Europe [14]. This raises the question as to the extent to which lifestyle factors such as obesity or severe weight gain, physical activity and diet before and during pregnancy [1, 41] play a role, irrespective of the methodological differences that exist between countries, as these could offer starting points for measures to prevent gestational diabetes. In Israel, for example, a nine-item questionnaire was developed using machine learning methods capable of assessing women’s risk of developing gestational diabetes already in early pregnancy [42]. Since the data set of inpatient obstetric quality assurance also contains information on maternal risk factors such as body mass index, weight gain during pregnancy or smoking, it could in future, after confirming the reliability of the relevant information, enable the use of innovative methods to detect women at increased risk of gestational diabetes, in line with the aforementioned study. Furthermore, the data set also facilitates the analysis of complications during birth depending on the presence of GDM.

## Limitations and strengths

The data basis of the present study includes all hospital births irrespective of the mothers’ insurance status and covers 97% of all births in Germany. Births outside of hospitals are not included, although the 2018 estimates for the prevalence of GDM here are significantly lower (1.3%) [43]. The selection bias for this study can be assumed to be lower than in cohort studies and analyses of claims data. Maternity records are the central source of information and have not only been documenting diagnosed cases of GDM, but also information on the results of pre-tests and diagnostic tests in the two-step screening procedure for GDM since 2014. Screening examinations will also document when a person does not have a test, whereas GDM tests will only document a positive result. In the maternity records of 90% of women who gave birth in hospital, whether they have received at least one of the two tests and with a positive or negative result is recorded. However, inconsistent documentation such as a positive diagnostic test without the diagnosis of GDM indicates limitations in the completeness and accuracy of the documentation. Thus, the proportion of women with a positive diagnostic test is 1 to 1.5 percentage points higher than the proportion of women with documented GDM and so an underestimation of GDM is therefore possible.

## Conclusion

The data of inpatient obstetric quality assurance appears to be a suitable source for continuous surveillance of the development of GDM screening participation and prevalence in Germany. Over time, there is an increase in the documented prevalence of GDM. In addition to the increase in the screening participation, changes in documentation behaviour, updates to diagnostic criteria implemented in 2012 and an increase in maternal risk factors may have contributed. More in-depth analyses should focus on the problems of incomplete documentation and inconclusive values found in two-step GDM screening. A further important question is whether women who have not yet been reached by screening differ with regard to important risk factors for GDM from women who have participated. This should allow validated assessments of the extent and causes of the increasing prevalence of GDM, which is observed in Germany as well as internationally. With regard to prevention measures, future analyses based on the described data set can, in addition to methodological differences, more strongly include maternal risk factors before and during pregnancy and take GDM-related complications into account.

## Key statements

- Maternity records show that the proportion of women who gave birth in hospital without a screening for gestational diabetes during pregnancy decreased from 16.6% in 2016 to 10.1% in 2018.
- 18.2% of women who gave birth in hospital had a pre-test and diagnostic test for gestational diabetes, 65.0% a pre-test only and 6.7% a diagnostic test only in 2018.
- 50,000 cases of gestational diabetes were documented in Germany among women with hospital births in 2018.
- In relation to all women who gave birth in hospital, the prevalence of documented gestational diabetes in Germany rose steadily from 4.6% in 2013 to 6.8% in 2018.

## Figures and Tables

**Table 1:** Description of the study population – women with hospital births (n=4,303,532) Source: External inpatient quality assurance for obstetrics at IQTIG, own calculations

	2013		2014		2015		2016		2017		2018	
	n	%	n	%	n	%	n	%	n	%	n	%
**Study population**	**652,479**	**100**	**684,163**	**100**	**707,995**	**100**	**752,040**	**100**	**754,082**	**100**	**752,773**	**100**
**Age group** <20 years 20–24 years 25–29 years 30–34 years 35–39 years 40–44 years ≥45 years	14,508 79,407 184,419 227,597 119,093 26,074 1,381	2.2 12.2 28.3 34.9 18.3 4.0 0.2	14,723 77,888 193,496 241,715 128,014 26,877 1,450	2.2 11.4 28.3 35.3 18.7 3.9 0.2	15,218 77,214 201,817 249,698 135,413 27,084 1,551	2.1 10.9 28.5 35.3 19.1 3.8 0.2	17,125 81,503 212,031 263,024 147,513 29,142 1,702	2.3 10.8 28.2 35.0 19.6 3.9 0.2	15,085 77,886 209,148 268,134 151,879 30,180 1,770	2.0 10.3 27.7 35.6 20.1 4.0 0.2	14,059 76,152 203,621 271,545 154,683 30,923 1,790	1.9 10.1 27.0 36.1 20.5 4.1 0.2

**Table 2:** Absolute and relative proportion of women who gave birth in hospitals and screening for gestational diabetes by test method, test result and reporting year (n=1,948,847) Source: External inpatient quality assurance for obstetrics at IQTIG, own calculations

Screening GDM	2016		2017		2018^1^	
	n	%	n	%	n	%
**Pre-test only^2^**	403,086		476,489		479,277	
Positive	11,556	2.9	14,004	2.9	13,629	2.8
Negative	391,530	97.1	462,485	97.1	465,648	97.2
**Diagnostic test only^3^**	53,369		46,449		49,280	
Positive	7,443	13.9	7,926	17.1	8,694	17.6
Negative	45,926	86.1	38,523	82.9	40,586	82.4
**Pre-test plus diagnostic test^3^**	170,812		135,570		134,515	
Positive	43,955	25.7	46,488	34.3	50,535	37.6
Negative	126,857	74.3	89,082	65.7	83,980	62.4

GDM = Gestational diabetes

^1^ In the reporting year 2018, 15,372 women with hospital births (2.0%) were excluded due to missing values (missings)

^2^ Test result refers to pre-test with 50g glucose (glucose challenge test)

^3^ Test result refers to diagnostic test with 75g glucose (oral glucose tolerance test, oGTT)

**Table 3:** Age-specific prevalence of documented gestational diabetes in women with hospital births by reporting year (n=4,303,532) Source: External inpatient quality assurance for obstetrics at IQTIG, own calculations

	2013		2014		2015		2016		2017		2018	
	n	%	n	%	n	%	n	%	n	%	n	%
**GDM diagnosis**	**29,735**	**4.6**	**31,400**	**4.6**	**36,016**	**5.1**	**40,065**	**5.3**	**45,632**	**6.1**	**51,318**	**6.8**
**Age group** <20 years 20–24 years 25–29 years 30–34 years 35–39 years 40–44 years ≥45 years	238 2,232 7,119 10,865 7,164 1,981 136	1.6 2.8 3.9 4.8 6.0 7.6 9.8	232 2,182 7,336 11,330 7,941 2,215 164	1.6 2.8 3.8 4.7 6.2 8.2 11.3	265 2,232 8,490 13,098 9,231 2,511 189	1.7 2.9 4.2 5.2 6.8 9.3 12.2	310 2,673 9,459 14,427 10,409 2,608 179	1.8 3.3 4.5 5.5 7.1 8.9 10.5	373 2,915 10,300 16,501 12,131 3,161 251	2.5 3.7 4.9 6.2 8.0 10.5 14.2	358 3,275 11,581 18,518 13,584 3,718 284	2.5 4.3 5.7 6.8 8.8 12.0 15.9

GDM = Gestational diabetes

**Table 4:** Overview of selected publications on the prevalence of gestational diabetes in Germany Source: Own table

Source	Data source	Study population	Definition GDM	Number of cases	Period	GDM prevalence
Bühling et al. [29]	Survey and examination data from the University Women’s Hospital Berlin	Women giving birth at the University Women’s Hospital without pre-existing diabetes	Two-step test procedure Screening with 50g CGT Diagnosis with 75g oGTT	N=1,416	1994–1996	8.2%
Festa et al. [30]	Survey and examination data of the Rudolfstiftung Hospital	Pregnant women in weeks 24 to 28 of gestation	Two-step test procedure Screening with 1h 75g Diagnosis with 75g oGTT	N=1,621	2001^*^	6.0%
Huy et al. [9]	Survey and examination data from the German Health Interview and Examination Survey for Children and Adolescents (KiGGS)	Mothers of participating children and young people	Information provided by mothers during the interview	N=2,970	2003–2006	5.3%
Domanski et al. [8]	Survey and examination data from the Survey of Neonates in Pomerania (SNIP) study	Mothers of newborns	Two-step test procedure Screening for glucosuria Diagnosis with 75g oGTT	N=5,801	2002–2008	5.1%
Reeske et al. [32]	AOK Berlin claims data	AOK-insured persons in Berlin with at least one year of insurance and pregnancy, excluding multiple pregnancies, multiple pregnancies within the study period, miscarriages and stillbirths, ectopic pregnancies and other diagnoses.	ICD-10 diagnosis: O24.4 without the presence of diabetes or O24.0–O24.3	N=3,338	2005–2007	16.0%
Beyerlein et al. [35]	Data of the inpatient quality assurance obstetrics in Bavaria	Women with hospital birth	Entry in the maternity record	N=81,129 N=92,589	2008 2014	3.4% 4.0%
Tamayo et al. [10]	Outpatient claims data of KV Nordrhein	SHI-insured persons in KV Nordrhein with pregnancy in at least one quarter	ICD-10 diagnosis: O24.4 without presence of E10–E14 or O24.1–O24.3	N=153,302 N=158,839	2012–2013 2013–2014	6.0% 6.8%
Melchior et al. [12]	Outpatient claims data of all KVs in Germany	SHI-insured persons nationwide with pregnancy in at least three quarters and no diabetes in two quarters before (ICD-10 diagnosis: E10–E14 or O24.0–O24.3)	ICD-10 diagnosis: O24.4, O24.9	N=567,191	2014–2015	13.2%
KBV [34]	Outpatient claims data of all KVs in Germany	SHI-insured persons nationwide with pregnancy in at least three quarters and no diabetes in two quarters before (ICD-10 diagnosis: E10–E14 or O24.0–O24.3)	ICD-10 diagnosis: O24.4 or O24.9	N=555,778 N=575,699 N=594.438	2015 2016 2017	12.9% 13.5% 13.9%
Reinders et al. [33]	Techniker Krankenkasse claims data	TK-insured persons with childbirth in the reporting year and with continuous insurance one year before pregnancy. Pregnancy of at least 20 weeks and GDM test (EBM 01776 or 01777).	ICD-10 diagnosis: O24.4 without presence of diabetes in the previous year (ICD-10 diagnosis: E10, E11 or ATC code 10A)	N=74,433	2016	14.7%
AQUA Institute (until 2014); IQTIG (from 2015) [11, 31]	Data from the nationwide inpatient quality assurance in obstetrics at the AQUA Institute or IQTIG	Women giving birth in hospital	Entry in the maternity record	N~650,000 N~650,000 N~650,000 N~650,000 N=658,201 N=638,798 N=650,232 N=638,951 N=651,696 N=658,735 N=690,547 N=714,574 N=758,614 N=761,176	2004 2005 2006 2007 2008 2009 2010 2011 2012 2013 2014 2015 2016 2017	2.2% 2.3% 2.4% 2.7% 3.4% 3.4% 3.7% 4.4% 4.3% 4.4% 4.5% 5.0% 5.4% 5.9%
Present study	Data from the nationwide inpatient quality assurance of obstetrics at IQTIG	Women with hospital birth without pre-existing diabetes	Entry in the maternity record or ICD-10 diagnosis O24.4 at discharge of inpatient stay	N=652,479 N=684,163 N=707,995 N=752,040 N=754,082 N=752,773	2013 2014 2015 2016 2017 2018	4.6% 4.6% 5.1% 5.3% 6.1% 6.8%

^*^ Year of publication, as observation period not specified

AQUA Institute = Institute for Applied Quality Improvement and Research in Health Care, AOK = Allgemeine Ortskrankenkasse, ATC = Anatomical Therapeutic Chemical Classification System, CGT = Glucose Challenge Test, EBM = Einheitlicher Bewertungsmaßstab, GDM = Gestational Diabetes, ICD = International Statistical Classification of Diseases and Related Health Problems, IQTIG = Institute for Quality Assurance and Transparency in Health Care, KV = Association of Statutory Health Insurance Physicians, KBV = National Association of Statutory Health Insurance Physicians, oGTT = oral glucose tolerance test, SHI = Statutory health insurance, TK = Techniker Krankenkasse

## Appendix

**Annex Table 1:** Comparison of the number of births in the IQTIG inpatient obstetric quality assurance dataset and the Federal Statistical Office Source: Federal Statistical Office – Statistics on births [26], External inpatient quality assurance for obstetrics at IQTIG, own calculations

	2013	2014	2015	2016	2017	2018
**Federal Statistical Office**						
Live-born	682,069	714,927	737,575	792,141	784,901	787,523
Stillborn	2,556	2,597	2,787	2,914	3,003	3,030
Multiples Twins Triplets Higher grade multiples	12,355 12,119 230 6	13,270 12,977 282 11	13,637 13,368 258 11	14,635 14,371 258 6	14,712 14,415 287 10	14,365 14,099 260 6
Estimated births^1^	672,028	703,950	726,445	780,150	772,885	775,916
**Inpatient quality assurance obstetrics**						
Births^2^	652,479	684,163	707,995	752,040	754,082	752,773
Difference Absolutely Relatively	19,549 2.9%	19,787 2.8%	18,450 2.5%	28,110 3.6%	18,803 2.4%	23,143 3.0%

IQTIG = Institute for Quality Assurance and Transparency in Health Care

^1^ Total live births and stillbirths – (number of twins + twice number of triplets + three times number of higher-grade multiples)

^2^ Women with pre-existing diabetes mellitus are excluded

**Annex Table 2:** Age-specific screening participation of pregnant women with hospital births by test procedure and reporting year (n=2,243,518) Source: External inpatient quality assurance for obstetrics at IQTIG, own calculations

	2016		2017		2018*			2016		2017		2018*	
	n	%	n	%	n	%		n	%	n	%	n	%
**Pre-test only**	**403,086**	**53.6**	**476,489**	**63.2**	**479,277**	**65.0**	**Pre- and diagnostic test**	**170,812**	**22.7**	**135,570**	**18.0**	**134,515**	**18.2**
**Age group**							**Age group**						
<20 years	8,826	51.5	9,389	62.2	8,977	65.6	<20 years	2,686	15.7	1,724	11.4	1,594	11.6
20-24 years	43,601	53.5	49,757	63.9	49,726	66.8	20-24 years	15,545	19.1	11,186	14.4	10,555	14.2
25-29 years	117,361	55.4	136,814	65.4	134,381	67.3	25-29 years	45,749	21.6	35,084	16.8	33,941	17.0
30-34 years	142,566	54.2	170,695	63.7	174,591	65.6	30-34 years	61,083	23.2	49,137	18.3	49,215	18.5
35-39 years	76,039	51.5	91,981	60.6	93,631	61.8	35-39 years	37,101	25.2	31,000	20.4	31,398	20.7
40-44 years	13,985	48.0	16,969	56.2	17,105	56.6	40-44 years	8,117	27.9	6,946	23.0	7,319	24.2
≥45 years	708	41.6	884	50.0	866	49.5	≥45 years	531	31.2	493	27.9	493	28.2
**Diagnostic test only**	**53,369**	**7.1**	**46,449**	**6.2**	**49,280**	**6.7**	**No test**	**124,772**	**16.6**	**95,570**	**12.7**	**74,329**	**10.1**
**Age group**							**Age group**						
<20 years	813	4.7	532	3.5	483	3.5	<20 years	4,800	28.0	3,439	22.8	2,630	19.2
20–24 years	4,538	5.6	3,458	4.4	3,437	4.6	20–24 years	17,819	21.9	13,485	17.3	10,676	14.4
25–29 years	13,909	6.6	11,045	5.3	11,313	5.7	25–29 years	35,012	16.5	26,205	12.5	19,984	10.0
30–34 years	19,554	7.4	17,469	6.5	18,711	7.0	30–34 years	39,821	15.1	30,833	11.5	23,806	8.9
35–39 years	11,944	8.1	11,324	7.5	12,419	8.2	35–39 years	22,429	15.2	17,574	11.6	13,960	9.2
40–44 years	2,462	8.4	2,473	8.2	2,736	9.1	40–44 years	4,578	15.7	3,792	12.6	3,062	10.1
≥45 years	149	8.8	148	8.4	181	10.3	≥45 years	313	18.4	242	13.7	211	12.1

^*^ In the reporting year 2018, 15,372 women with hospital births (2.0%) were excluded due to missing values (missings)

**Annex Table 3:** Proportion of positive screening tests by testing method used, reporting year and age of women at birth (n=1,948,847) Source: External inpatient quality assurance for obstetrics at IQTIG, own calculations

	Pre-test only^1^			Diagnostic test only^2^			Pre- and diagnostic test^2^		
	2016	2017	2018*	2016	2017	2018*	2016	2017	2018*
**Age group**	**in percent**								
<20 years 20–24 years 25–29 years 30–34 years 35–39 years 40–44 years ≥45 years	2.1 2.5 2.6 2.8 3.4 4.2 4.4	2.0 2.7 2.6 2.9 3.5 4.4 6.1	2.2 2.3 2.6 2.8 3.3 4.7 6.7	7.9 10.4 11.7 13.6 17.2 21.7 26.0	11.4 13.5 14.9 16.5 19.8 24.1 25.8	9.9 13.8 15.8 16.6 20.5 25.6 26.5	14.7 19.3 22.6 25.4 31.0 36.9 44.4	21.7 26.9 30.3 33.7 40.0 46.7 51.7	23.4 30.0 33.6 36.9 43.1 50.0 56.6
**Total**	**2.9**	**2.9**	**2.8**	**13.9**	**17.1**	**17.6**	**25.7**	**34.3**	**37.6**

^1^ Test result refers to pre-test with 50g glucose (glucose challenge test, GCT)

^2^ Test result refers to diagnostic test with 75g glucose (oral glucose tolerance test, oGTT)

^*^ In the reporting year 2018, 15,372 women with hospital births (2.0%) were excluded due to missing values (missings)
